# Detection of Recent Myocardial Infarction Using Native T1 Mapping in a Swine Model: A Validation Study

**DOI:** 10.1038/s41598-018-25693-1

**Published:** 2018-05-09

**Authors:** Chen Cui, Shuli Wang, Minjie Lu, Xuejing Duan, Hongyue Wang, Liujun Jia, Yue Tang, Arlene Sirajuddin, Sanjay K. Prasad, Peter Kellman, Andrew E. Arai, Shihua Zhao

**Affiliations:** 1 Department of Magnetic Resonance Imaging, Fuwai Hospital, State Key Laboratory of Cardiovascular Disease, National Center for Cardiovascular Diseases, Chinese Academy of Medical Sciences and Peking Union Medical College, Beijing, China; 2 Department of Pathology, Fuwai Hospital, State Key Laboratory of Cardiovascular Disease, National Center for Cardiovascular Diseases, Chinese Academy of Medical Sciences and Peking Union Medical College, Beijing, China; 3 Department of Animal Experimental Center, Fuwai Hospital, State Key Laboratory of Cardiovascular Disease, National Center for Cardiovascular Diseases, Chinese Academy of Medical Sciences and Peking Union Medical College, Beijing, China; 4 National Heart, Lung and Blood Institute (NHLBI), National Institutes of Health (NIH), Bethesda, Maryland USA; 5 NIHR Cardiovascular Biomedical Research Unit, Royal Brompton & Harefield NHS Foundation Trust, London, UK; 6 Cardiovascular and Pulmonary Branch, National Heart, Lung and Blood Institute, National Institutes of Health, US Department of Health and Human Services, Bethesda, MD USA

## Abstract

Late gadolinium enhancement (LGE) imaging is the currently the gold standard for *in-vivo* detection of myocardial infarction. However, gadolinium contrast administration is contraindicated in patients with renal insufficiency. We aim to evaluate the diagnostic sensitivity and specificity of this contrast-free MRI technique, native T1 mapping, in detecting recent myocardial infarction versus a reference histological gold standard. Ten pigs underwent CMR at 2 weeks after induced MI. The infarct size and transmural extent of MI was calculated using native T1 maps and LGE images. Histological validation was performed using triphenyl tetrazolium chloride (TTC) staining in the corresponding *ex-vivo* slices. The infarct size and transmural extent of myocardial infarction assessed by T1 mapping correlated well with that assessed by LGE and TTC images. Using TTC staining as the reference, T1 mapping demonstrated underestimation of infarct size and transmural extent of infarction. Additionally, there was a slight but not significant difference found in the diagnostic performance between the native T1 maps and LGE images for the location of MI. Our study shows that native T1 mapping is feasible alternative method to the LGE technique for the assessment of the size, transmural extent, and location of MI in patients who cannot receive gadolinium contrast.

## Introduction

Myocardial infarction (MI) remains a major cause of premature morbidity and mortality. Myocardial scar tissue resulting from MI is associated with ventricular remodeling^1,2^, risk of ventricular tachycardia^3^ and sudden cardiac death^4^. Thus, comprehensive evaluation of the location and transmural extent of post-MI scar tissue is important for clinical management and risk stratification.

Currently, late gadolinium enhancement (LGE) imaging acquired during cardiovascular magnetic resonance (CMR) approximately 10 minutes after the administration of gadolinium contrast is considered the *in-vivo* gold standard for detection and quantification of myocardial infarction^5,6^. However, this technique has several important limitations. Administration of gadolinium contrast is contraindicated in patients with acute and chronic renal insufficiency. However, patients with renal insufficiency represent approximately one fifth of patients presenting with MI^7^. There is also a small risk of adverse reactions to the gadolinium contrast^8^. Finally, post-MI patients with a higher New York Heart Association Class often have orthopnea^9^. CMR imaging with LGE is long in duration, and it can be challenging to image this group of patients with this technique.

Native T1 mapping is a novel technique that can perform quantitative characterization of the myocardium without the need of a gadolinium contrast agent. Thus, T1 mapping may be an appropriate and safe alternative technique for MI patients who also have renal insufficiency. Unlike LGE imaging, the signal intensity in the T1 map is derived from the absolute T1 relaxation time^10,11^ (in milliseconds), which is an excellent parameter for quantitative assessment.

During an acute MI, cellular edema from ischemia results in increased signal intensity in the area of infarction on both LGE and T2 weighted images^12^. In chronic MI, the area of infarction is replaced with fibrosis, resulting in an area of increased extracellular volume that is well depicted as an area of increased signal intensity on LGE images. In both acute and chronic MI, the T1 value on native T1 maps is also increased^13^. Using LGE as a reference standard, previous studies have demonstrated that native T1 maps can detect the change of the T1 signal in the infarcted area of myocardium at 1.5 T^14,15^ and 3T^16^. In addition, native T1 maps can be used to determine the size and transmural extent of infarction in both acute^15^ and chronic MI^17^ patients. Similar results have been validated in canine models at 3T^13^ as well. However, a key limitation of current work is the lack of direct histological validation.

The aim of this study is to determine the diagnostic accuracy, sensitivity and specificity of *in-vivo* native T1 mapping in determining infarct location, size and transmural extent compared to *in-vivo* LGE images and *ex-vivo* histopathology in a swine model.

## Methods

### Animal model

Approval was obtained from the Ethics Committee for Animal Study in Fuwai Hospital and the Care of Experimental Animals Committee of the Chinese Academy of Medical Sciences and Peking Union Medical College to prospectively study twelve Chinese mini-pigs weighing approximately 28–32 kg. A midline sternotomy was performed, and the left anterior descending coronary artery was occluded for 90-minutes to produce MI. All animals received antimicrobial therapy (cephazolin 1.0 g, intramuscular injection, twice daily for 3 days) and buprenorphine (0.3 mg, twice daily for 3 days) for postoperative analgesia. This study conformed with the ‘Guide for the Care and Use of Laboratory Animals’ published by the US National Institutes of Health (NIH Publication No. 85-23, revised 1996) and the ‘Regulation to the Care and Use of Experimental Animals’ of the Beijing Council on Animal Care (1996).

### CMR studies

All CMR examinations were performed on a 1.5 T MR scanner (Avanto®, Siemens Medical Systems) with a high-performance gradient system, an eight-element array body surface coil, and six-element spine coil. A three-lead ECG was used for gating. The animals underwent MRI examination two weeks (14 ± 2 days) after the MI and were anesthetized prior to the scan using sodium pentobarbital (25 mg/kg).

An ECG-gated steady-state free precession cine sequence with time-adaptive sensitivity encoding technique^18^ was used to obtain volumetric short-axis cine images through the left ventricle (6–10 slices without gaps) as well as four and two-chamber long axis images of the left ventricle. Subsequently, T1 modified look-locker inversion-recovery (MOLLI) images of three short axis slices (basal, mid and apical) of the left ventricle were obtained prior to the administration of a gadolinium contrast agent^15,19,20^. The apical short axis slice was defined as the slice immediately adjacent to the apical cap, the mid-ventricular short axis slice was defined as the slice positioned at the middle of papillary muscles. The basal short axis slice was defined as the last slice to show intracavitary blood pool on cine imaging. The MOLLI acquisition had a total of two inversions: 3 images were acquired after the first inversion which was followed by a pause of 3 heart beats, and then 5–6 images were acquired after a second inversion. Typical imaging parameters of the MOLLI acquisition were: field of view of 360 × 250 mm^2^, matrix of 134 × 256, slice thickness of 6 mm, in-plane resolution of 1.4 × 1.4 mm^2^, time to repetition/time to echo of 2.68/1.13 ms, flip angle of 35◦, acquisition window of 287 ms, and parallel acquisition technique factor of 2.

Approximately 10–15 min after a 0.2 mmol/kg intravenous dose of gadopentetate dimeglumine (Magnevist, Bayer Healthcare Pharmaceuticals, Wayne, NJ USA), LGE phase-sensitive inversion-recovery gradient-echo pulse sequence was used to acquire images at the same sectional location as the cardiac cine images^21^. Detailed LGE imaging parameters were as follows: field of view of 260 × 320 mm^2^, matrix of 208 × 206, slice thickness of 6 mm, in-plane resolution of 1.25 × 1.25 mm^2^, time to repetition/time to echo of RR interval/3.26 ms, flip angle of 35, acquisition window of 168 ms, parallel acquisition technique factor of 2.

The total scan time of the T1 mapping and LGE imaging were about 40 s (12–14 s per slice) and 1 to 1.5 min (8–10 s per slice) respectively. The average CMR scan time was 22.4 ± 5.2 min.

### Histological validation

Upon completion of the MRI scan, the animals were euthanized via intravenous injection of 20 ml 15% (v/w) potassium chloride under anesthesia. The explanted hearts were fixed by immersion in 95% ethanol for 20 min, precooled to −80 °C, and sectioned from base to apex into 6-mm-thick short-axis slices by an experienced slicer. The slices were then immersed in a 1% triphenyl tetrazolium chloride (TTC) solution in saline at 37 °C for 10 min, and digitally photographed.

### Image analysis

Landmarks such as papillary muscles and right ventricular insertion points were used to match the TTC images to the CMR images. This process was performed by a radiologist and a pathologist by consensus.

The LGE images and T1 maps were analyzed on CVI 42 image analysis software (Circle Cardiovascular Imaging Inc, Calgary, Canada). The remote area (normal viable myocardium used as reference) of each slice was identified as a region with no hyperintensity on LGE and no elevated T1 value on the T1 maps. A reference region of interest was drawn in the remote area on both the LGE images and T1 maps by two experienced radiologists independently. The infarcted myocardium was identified automatically by the software as a region with a mean signal intensity >5SDs^22^ relative to remote myocardium on LGE images and a mean T1 value >3SDs on the T1 maps^15,17^ relative to the remote myocardium. The software automatically calculated the size of the infarcted area on both the LGE images and the T1 maps. The infarct size on *ex-vivo* TTC staining images was measured using the software of image J (version 1.45 s, National Institute of Health, Bethesda, USA). The infarct size assessed by the T1 maps, LGE images and TTC images, were measured as the percentage of the slice area.

Analysis of the transmural extent of the infarcted area was determined by assessing the average extension of infarct from the endocardium toward the epicardium on each slice. Ten equidistant transmural radii were drawn perpendicular from the endocardial to the epicardial boundaries within infarct area. The transmural extent of the corresponding slice was defined as the averaged percentage extent of infarct along the radii^23^.

To assess the reproducibility of infarct size and transmural extent measurement on the T1 map, the measurement of infarct size and transmurality were performed by 2 experienced observers (with 14 and 5 years of image analysis experience) independently.

For the segmental level analysis, the basal, midventricular and apical short-axis images were segmented in to 16 segments according to the American Heart Association 17-segment model (the apical cap was not included). The average T1 value of each segment was measured and the presence or absence of infarction on LGE and TTC images was recorded.

Functional analysis was performed using the software of CVI 42 applying the Simpson rule in short-axis cines. The endo- and epicardial contours were semiautomatic detected with manual correction. Papillary muscles and myocardial trabeculations were included in the ventricular cavity. End diastole and end systole was defined as the phase of cardiac cycle with the largest and smallest ventricular cavity respectively^24^.

### Statistical analysis

Statistical analyses were performed using MedCalc Statistical Software version 16.2.0 (MedCalc Software bvba, Ostend, Belgium). The Shapiro-Wilk test and quantile-quantile plots were used to assess for normal distribution of the data. The T1 values of the remote and infarcted myocardium were compared using either student’s t test or Wilcoxon signed-rank test depending on whether or not the data was normally distributed. Simple linear regression was performed to estimate the correlation between the T1 and LGE techniques in respect to measurement of infarct size and transmural extent. Agreement of the infarct size and transmural extent in each animal measured by the T1 and LGE techniques was performed using Bland-Altman analysis. Differences in the infarcted size and transmural extent measured on the LGE image, T1 map and TTC image were analyzed by randomized block ANOVA, followed by Dunnett’s t-test. Inter-observer reproducibility of infarct size and transmural extent was performed using Bland-Altman analysis.

ROC analysis was performed to evaluate the performance of T1 mapping and LGE imaging in detecting the presence of MI as well as to identify the best cut-off value for T1 mapping to detect MI using TTC as the gold standard. Specifically, all the segments in the T1 maps and LGE images were categorized as TTC+ or TTC− according to the presence or the absence of infarction in corresponding TTC segments. Segments with MI on TTC staining were defined as the “true positive”; segments without MI on TTC staining were defined as the “true negative” in the analysis. When generating the ROC curves, the T1 values were used as continuous quantitative data and the LGE results were used as dichotomous data. The comparison of areas under the ROC curves was performed using the method of Delong *et al*.^25^ and the best-cut value was determined by Youden’s Index.

The datasets generated and analyzed during the current study are available from the corresponding author on reasonable request.

## Results

### Mortality and image analysis

Of the 12 animals that had the coronary artery occlusion, one pig did not survive during the procedure and one died one day after the MI induction (death rate of 16.7%). This left a total of 10 pigs for analysis. In the remaining 10 pigs, induced MI was verified by the presence of hyperintensity on the LGE images before the animal was sacrificed. The MRI and TTC images were successfully obtained in the 10 remaining pigs. All 10 pigs that underwent MRI scan were LGE positive (100%). On the TTC images, the average number of slices with MI was 5 ± 0.6, and the average number of AHA-segments to have MI was 4.5 ± 1.3.

**Figure 1:**
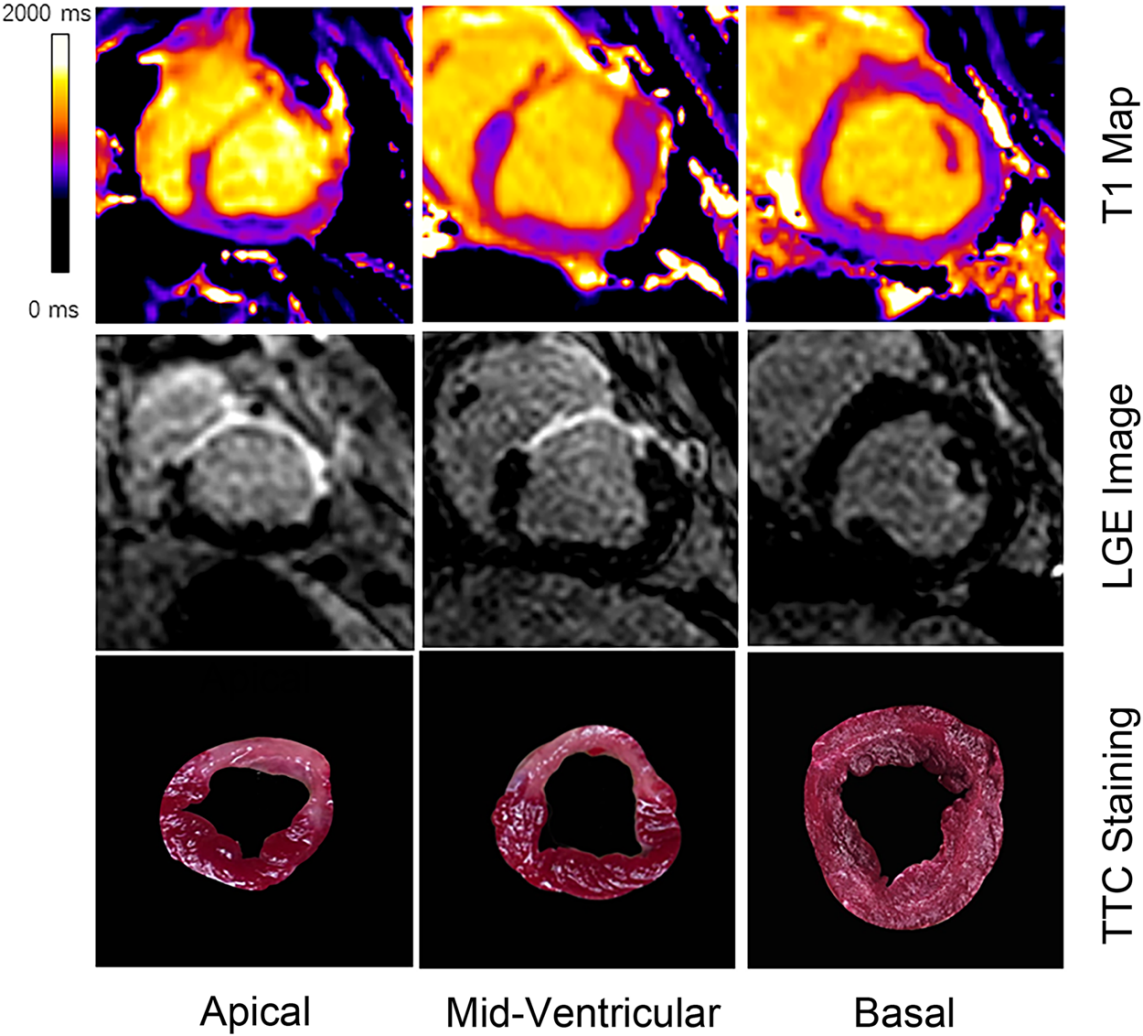
Detection of MI on T1 maps, LGE images and TTC images. Legend: Representative T1 map, LGE image, and TTC image of basal, mid-ventricular and apical slices acquired 2 weeks after MI in a swine model. Infarcted myocardium is detected at the same location on the three different types of images.

Thirteen of the matched 30 slices in the 10 pigs were excluded. Specifically, 10 basal slices had no MI and were not analyzed. One mid-ventricular slice was excluded because of significant motion artifacts on the corresponding LGE image and one apical slice was excluded because of susceptibility artifacts present on the T1 map rendering it non-interpretable. Another apical slice was excluded due to the poor match with the corresponding TTC image. Seventeen matched slices were used in the final study. Representative images are shown in the Fig. 1. The entire list of matched images can be found as Supplementary Figure S1.

### CMR findings

#### Left ventricular function

The mean indexed left ventricular end-diastolic volume and mean indexed end-systolic volume of the pigs was 59.3 ± 8.3 ml/m^2^ and 35.4 ± 7.3 ml/m^2^ respectively. The mean left ventricular ejection fraction was 40.5 ± 8.2% and the mean indexed myocardial mass was 49.3 ± 8.5 g/m^2^.

#### Infarct size and transmural extent detected by T1 mapping

**Figure 2:**
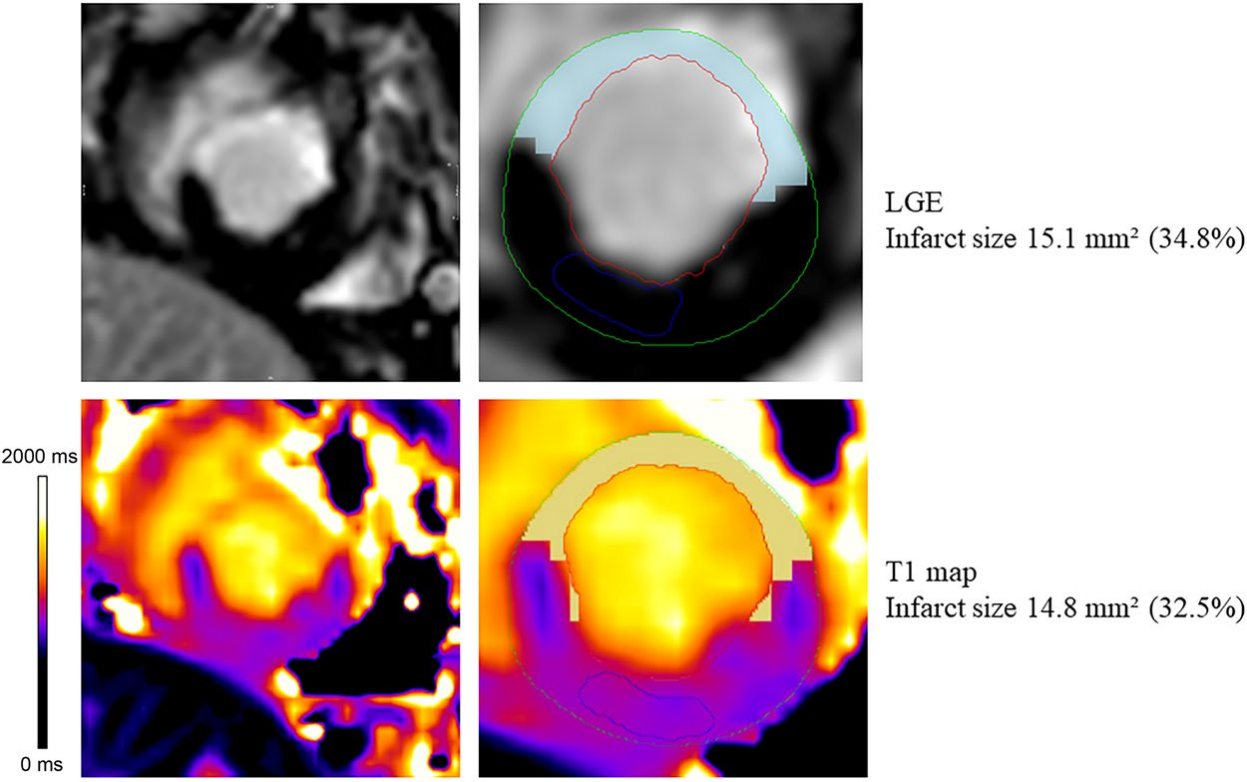
Infarct size analysis in a swine MI model at 1.5 T. Legend: Infarcted myocardium is identified on both images using the mean + 5 SD criterion for the LGE image and mean + 3 SD criterion for the T1 map (highlighted in blue and yellow pixels on the LGE image and T1 map) in respect to a reference region of interest drawn in the remote myocardium (blue contours). The infarcted area accounts for 32.5% and 34.8% of the entire myocardium of the slice on the T1 map and the LGE image respectively.

The software program successfully identified the infarcted myocardium on both the LGE images and the T1 maps (Fig. 2). The average absolute infarct area on the T1 maps, LGE images and TTC images were 90.6 ± 32.5 mm^2^, 94.2 ± 35.5 mm^2^ and 102.8 ± 35.5 mm^2^, respectively. The average percentages of infarct on the T1 maps, LGE images and TTC images of each myocardium slice were 25.5 ± 10.5%, 26.5 ± 11.1% and 28.9 ± 11.6% respectively. Compared with TTC staining, T1 maps significantly underestimated the infarct size (p < 0.001 for both absolute and percentage of infarct area). However, no significant difference was found between the infarcted area assessed by the T1 maps and the LGE images (p = 0.207 for absolute infarct area, p = 0.155 for percentage infarct area).

**Figure 3:**
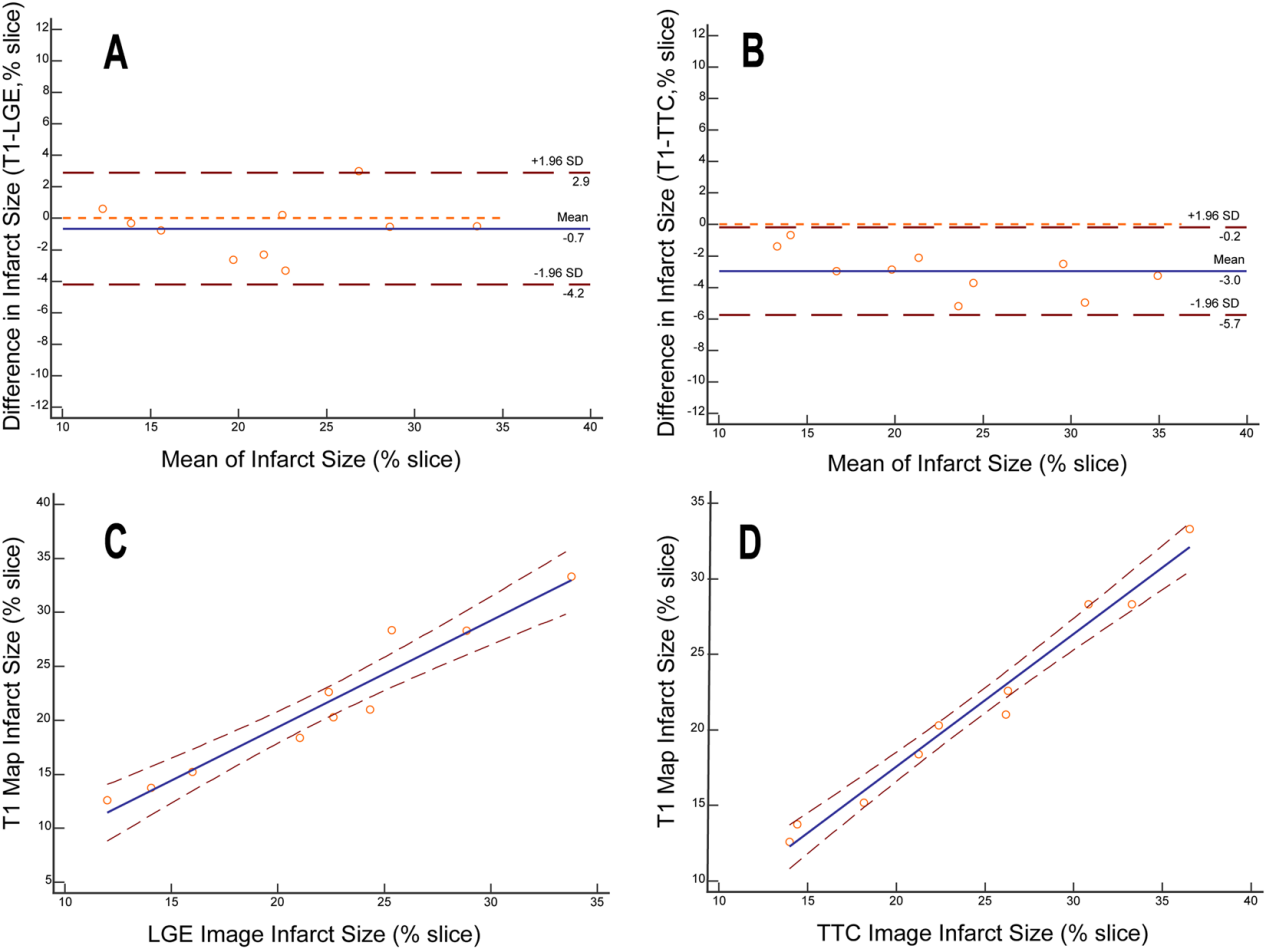
Infarct size assessed by T1 mapping, LGE imaging and TTC staining. Legend: Bland-Altman analysis shows good agreement in the measurement of infarct size between the T1 maps and LGE images (**A**) as well as between the T1 maps and TTC staining images (**B**). The blue line indicates the mean of infarct size. The dashed red line indicates 95% CI. There is good correlation between the LGE images and T1 maps, y = −0.41% + 1.00x, R^2^ = 0.93, p < 0.0001, (**C**) as well as between the TTC staining images and the T1 maps, y = −0.02% + 0.88x, R^2^ = 0.98, p < 0.0001, (**D**) in measuring the infarct size.

Bland-Altman analysis showed good agreement between the T1 maps and the LGE images for measuring the average infarct size in each animal (limits of agreement = −0.66 ± 1.80%, Fig. 3A); and there was moderate agreement in infarct size detected by T1 maps and TTC staining for each animal (limits of agreement = −2.95 ± 1.42%, Fig. 3B). The average infarct size of each animal assessed by T1 mapping correlated well with infarct size assessment by LGE (y = −0.41% + 1.00x, R^2^ = 0.93, p < 0.0001, Fig. 3C) and by TTC staining (y = −0.02% + 0.88x, R^2^ = 0.98, p < 0.0001, Fig. 3D).

**Figure 4:**
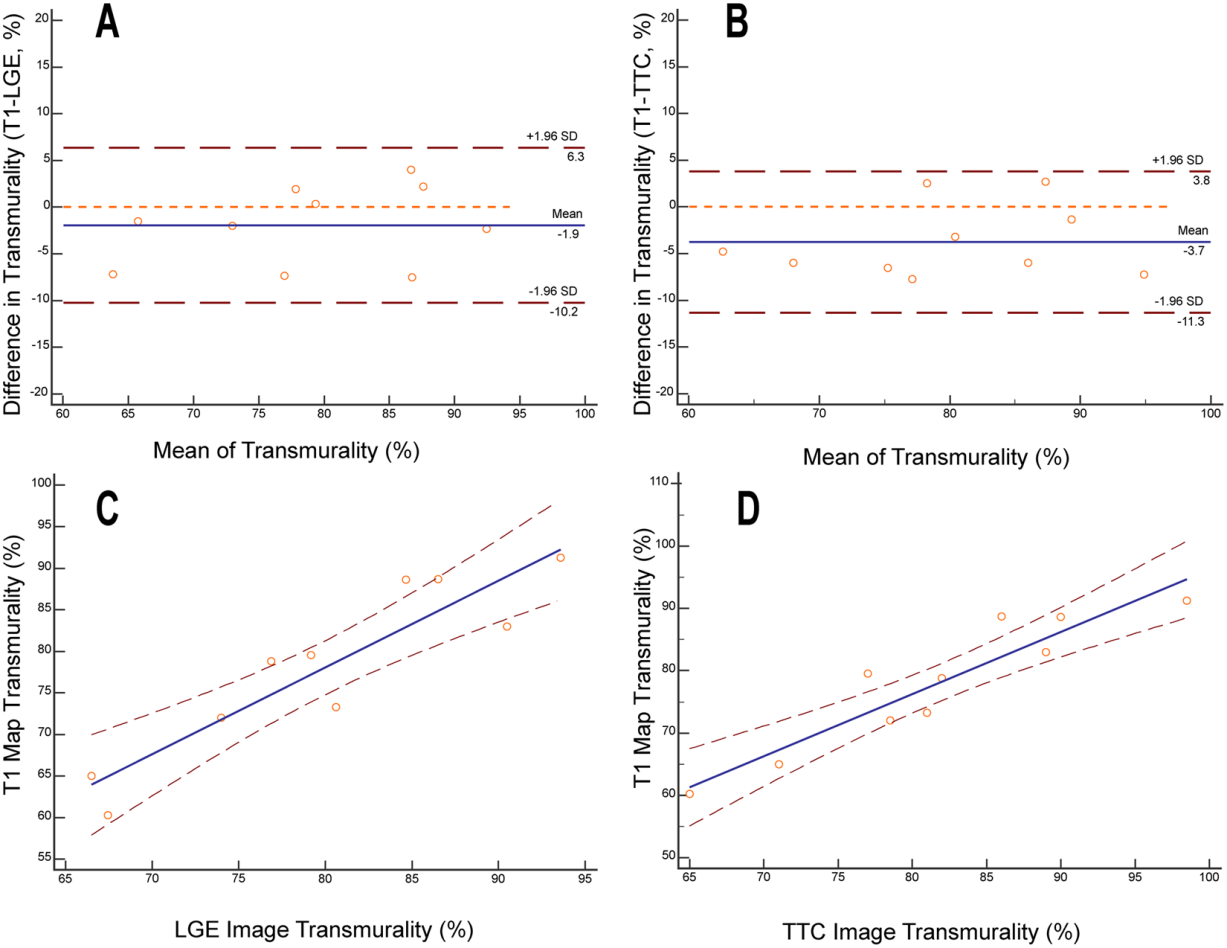
Transmural extent of myocardial infarction assessed by T1 mapping, LGE imaging and TTC staining. Legend: Bland-Altman analysis shows good agreement between the T1 maps and LGE images (**A**) as well as the T1 maps and TTC staining images (**B**) in regard to determining the transmural extent of myocardial infarction. The blue line indicates the mean of infarct size. The dashed red line indicates 95% CI. There is good correlation between LGE images and T1 maps, y = −5.61% + 1.05x, R^2^ = 0.84, p = 0.0002, (**C**) as well as TTC staining images and T1 maps, y = −3.39% + 1.00x, R^2^ = 0.86, p = 0.0001, (**D**) for determining the transmural extent of myocardial infarction.

The average transmural extent of the infarction assessed by T1 mapping, LGE imaging and TTC imaging was 78.1 ± 10.4%, 80.0 ± 9.1% and 81.8 ± 9.7% respectively. The average transmural extent assessed by the T1 maps was significantly smaller than that assessed by TTC images (p = 0.012). Bland-Altman analysis showed good agreement in average transmural infarct extent assessed by the T1 map and the LGE images (limits of agreement = −1.93 ± 4.22%, Fig. 4A). There was also good agreement in the average transmural extent of infarction assessed by the T1 map and the TTC image for each animal (limits of agreement = −3.74 ± 3.85, Fig. 4B). The average transmural extent of infarction for each animal assessed by T1 mapping correlated well with that assessed by LGE (y = −5.61% + 1.05x, R^2^ = 0.84, p = 0.0002, Fig. 4C) and TTC (y = −3.39% + 1.00x, R^2^ = 0.86, p = 0.0001, Fig. 4D).

#### Diagnostic performance of T1 mapping to detect MI at segmental level

**Figure 5:**
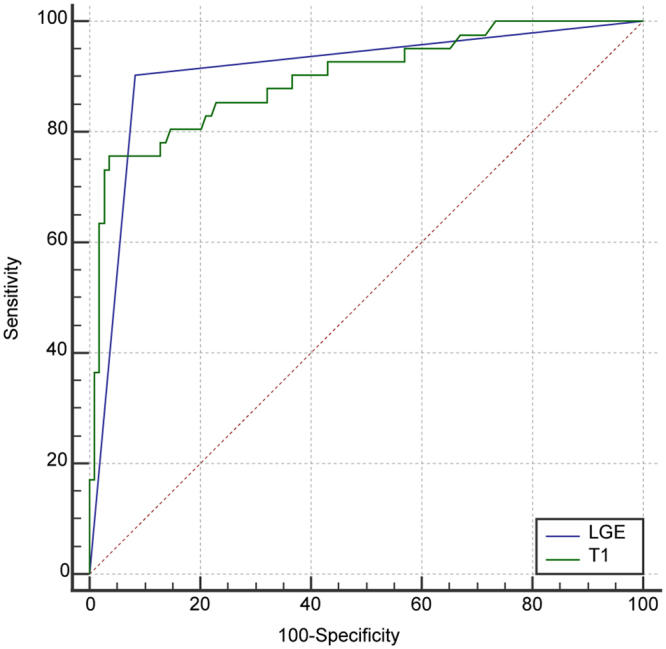
ROC analysis of the diagnostic performance of T1 mapping and LGE imaging in the detection of MI (using TTC staining as the gold standard). Legend: ROC analysis shows the diagnostic performance of T1 mapping compared to the LGE technique in detecting MI at segmental level against TTC staining as the gold standard. T1 mapping had an AUC of 0.89. The cut of value of 1124 ms had a highest Youden index (0.719), with a sensitivity of 75.6% and a specificity of 96.3%. There was no significant difference between the two techniques in AUC for the detection of MI.

Of the 160 possible segments from the basal, mid-ventricular and apical slices, one mid-ventricular slice (6 segments) and two apical slices (8 segments) were excluded for reasons previously described. Of the 146 available segments, TTC images showed MI in 41 segments. LGE images showed MI in 46 segments. Nine of the 46 LGE positive segments were determined to be false positives when compared to TTC images. Figure 5 illustrates the performance of T1 mapping and LGE imaging in detection of MI using TTC stained images as the gold standard based on ROC analysis. The area under the curve (AUC) was 0.89 ± 0.02 (95% CI: 0.84–0.94) for T1 mapping and 0.91 ± 0.02 (95% CI: 0.852 to 0.950) for LGE imaging. There was no significant difference between the two techniques to detect MI segments (z statistic: 0.31, p = 0.756). A T1 value of 1124 ms had the highest Youden index (0.719) with sensitivity of 75.6% and specificity of 96.3% to detect MI as defined by the TTC images, while the LGE images had sensitivity of 90.2% and specificity of 91.7%. Using 1124 ms as the cut-off value for MI segment detection, 35 segments on the T1 maps were labeled as MI positive, 4 of which were false positive when compared to the TTC images.

#### Tissue characteristics of infarcted myocardium

The T1 value and the LGE signal intensity characteristics of the infarcted and remote myocardium are summarized in Table 1. The average T1 value of infarcted area was 1347.7 ± 89.4 ms, which was significant higher than that of the remote area (995.0 ± 49.4 ms; p < 0.0001). The average difference between the T1 of infarcted and remote myocardium was 352.8 ± 107.8 ms. The mean variability of T1 value in infarcted segments and remote segments was 80.5 ± 33.1 ms and 47.9 ± 25.1 ms, respectively.

**Table 1:** Detailed T1 value and SI of LGE in remote and infarct area (averaged to each animal).

Animal Number	Mean/SD SI in Infarcted Myocardium on LGE Image	Mean/SD SI in Remote Myocardium on LGE Image	Normalized Difference in LGE SI	Mean/SD T1 of Infarcted Myocardium, ms	Mean/SD T1 of Remote Myocardium, ms	Normalized Difference in T1
1	115.8 ± 7.3	16.7 ± 5.05	627.4%	1406 ± 126	1021 ± 96	38.50%
2	135.6 ± 15.2	24.0 ± 7.9	465.4%	1261 ± 73	1030 ± 30	22.70%
3	84.9 ± 24.3	18..5 ± 11.8	358.1%	1486 ± 82	981 ± 44	51.40%
4	106.5 ± 11.4	22.7 ± 6.7	371.7%	1327 ± 70	1027 ± 41	29.30%
5	258.7 ± 22.0	31.6 ± 13.3	730.8%	1421 ± 83	966 ± 50	47.20%
6	105.9 ± 13.3	20.1 ± 8.65	439.2%	1349 ± 74	980 ± 41	37.60%
7	82.5 ± 17.8	11.5 ± 7.9	617.3%	1261 ± 71	1039 ± 36	21.37%
8	80.1 ± 19.7	14 ± 5.7	472.1%	1310 ± 77	1034 ± 37	26.69%
9	97.2 ± 18.6	19.9 ± 4.8	388.4%	1300 ± 89	942 ± 37	38.00%
10	105.0 ± 22.7	22.3 ± 9.2	371.0%	1271 ± 58	946 ± 52	47.50%
Average	112.6 ± 17	21 ± 8.4	482.6 ± 165.5%	1347 ± 89	995 ± 48	35.8 ± 11.7%

SI = signal intensity, SD = Standard Deviation.

Normalized Difference in LGE SI/T1 = (Infarcted_SI/T1_ − Remote_SI/T1_)/Remote_SI/T1_.

Regarding the infarcted to remote myocardium contrast, the percentage change of signal intensity on LGE images was 482.6 ± 165.5%, which was about 14-fold higher than that in T1.

#### Inter-observer reproducibility of assessment infarct size and transmural extent of infarction

**Figure 6:**
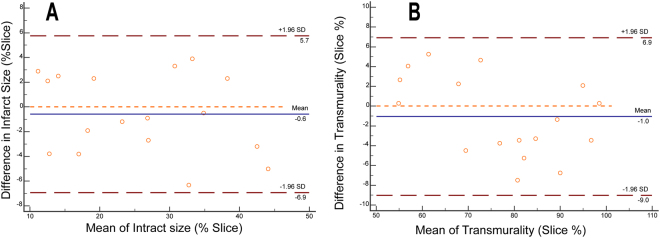
Bland-Altman analysis for Inter-observer reproducibility in infarct size and transmurality measurement Legend: Inter-observer reproducibility for measuring the infarct size (**A**) and transmurality (**B**). (The blue line indicates the mean of infarct size; The dashed red line indicates 95% CI).

Inter-observer reproducibility for measuring MI size and transmural extent of infarction on T1 maps was good. The limits of the agreement for assessing MI size was −0.53 ± 3.23%, and for determining transmural extent of infarction was −1.04 ± 4.07% (The Bland-Altman plots are presented in Fig. 6).

## Discussion

Our study provides histopathological validation that native T1 mapping has high specificity for MI detection at the segmental level. Although good correlation and inter-observer reproducibility were found, native T1 mapping underestimated the size and transmurality of MI at 1.5 T relative to histopathology.

The location, transmural extent and the size of an MI is closely related to the prognosis in both acute^26^ and chronic^27^ MI patients. LGE imaging is considered a powerful non-invasive imaging technique for MI detection^28,29^. However, the administration of gadolinium agents is contraindicated in patients with acute and chronic renal insufficiency, prohibiting the use of the LGE technique in this patient population. This is because of the risk of nephrogenic systemic fibrosis (NSF)^30^, which was first reported in 2000 in 15 dialysis patients^31^. As information surfaced showing a relationship between gadolinium administration in patients with renal insufficiency and NSF, the use of gadolinium contrast agents was restricted in the setting of renal impairment^32^. Unfortunately, approximately 20% of MI patients have renal insuffienciency^7^. These patients would greatly benefit from a contrast-free imaging technique that could assess myocardial infarction.

The T1 mapping technique measures the T1 value of the myocardium, which can be used for quantitative analysis. Comparability and reproducibility of T1 mapping across studies and patients is highly desirable as it does not rely on gadolinium contrast^33^. Additionally, T1 mapping can improve the MRI examination workflow by shortening the exam time, as the LGE requires a waiting period after gadolinium contrast administration before images can be obtained.

Assessment of MI with native T1 maps has previously been evaluated in animal models^17^ and patients^15,34^. However, none of the previous study have validated T1 maps using histology as a gold standard. Kali *et al*. have shown that native T1 can reliably determine the characteristics of MI in animal models at 3T when compared to an LGE reference standard using a mean + 5 SD as the criterion. However, they also found that T1 maps underestimate infarct size and transmural extent at 1.5 T^22^. Compared to previous studies^13,15^, we used a less stringent criterion (mean + 3 SD) for the T1 maps, which has been reported to have a better performance at 1.5 T^13^. However, using this criterion, we still found approximately a 10% underestimation in infarct size and 5% underestimation in transmural extent of infarction relative to histopathology, which is likely secondary to limited spatial resolution and relatively poor contrast between the infarcted and intact myocardium at 1.5 T. Nevertheless, our results show that T1 mapping determines the location of MI at the segmental level with high specificity.

Compared to LGE imaging, native T1 mapping has higher specificity and lower sensitivity. Native T1 mapping was slightly inferior to LGE imaging in identification of MI, however this different was not significant. Of note, we used continuous T1 values to generate an ROC curve rather than using the categorical data generated by a threshold because continuous values generate a smooth ROC curve which is better for comparison with the curve of LGE. In addition, the cut-off value we generated from the ROC curve, 1124 ms, was very close to the mean + 3 SD of T1 in the remote area, 1139 milliseconds. This further affirmed the ability of native T1 in detection of MI at segmental level.

The performance of T1 mapping for characterization of acute (within 7 days) and chronic MI have been previously validated^13,15,17,35^. Also, a previous study demonstrated that native T1-weighted images can be used to detect edema resulting from a recent MI^14^ (within 2 months after). The time point of 2 weeks that we used falls outside of the early stage of the acute phase of MI and may not fully simulate the acute ischemic injury. However, the predominant pathological change in the acute phase of an MI is myocardial edema and this change lasts up to 40 days^36^.

Dall ‘Armellina *et al*. have shown that the increase of T1 is closely related to the rise of T2 in the acute phase following an MI^22,37^, and can be considered as a surrogate marker for edema. However, myocardial edema only partially explains the elevation of T1 value because the extent of edema decreases drastically in the first month after the onset of MI^38^ but myocardial T1 abnormality persists much longer. Mechanisms for T1 elevation in chronic MI^39,40^ may be associated with the change of T1 value in our study. However, since edema peaks within the first week and begins to resolve thereafter^38^, if measured at an earlier time point, the difference in T1 between infarcted and remote area may be more prominent and the characterization of MI may possibly be more reliable.

There are still several technical limitations for clinical application of native T1 mapping in MI. A perfect delineation of scar area can be challenging when spatial resolution is insufficient and the most common T1 mapping sequence used in clinic is sensitive to the variation of heart rate. Therefore, parameters need to be optimized during image acquisition and post processing^41^. In addition, the measured T1 value is prone to be influenced by acquisition scheme, field strength, vendors and numerous other factors^33^. These factors should be considered during the interpretation of the data.

We acknowledge the following limitations of our study. Firstly, the AUC of the T1 mapping to detect MI is smaller than that of LGE image, but most likely we did not have the sample size to find a difference. A larger study might be capable of showing that LGE is still overall superior to T1 mapping. Secondly, our study obtained only three representative native T1 left ventricular short axis slices for the apical, mid-ventricular and basal plane rather than volumetric coverage. This results in a smaller amount of data for the slice level analysis, and additional studies with volumetric T1 mapping coverage are necessary to evaluate the efficacy of this technique. Thirdly, we did not perform pixel-based analysis because of the limitations of the software. Future work should address this. Finally, the MR images and corresponding TTC staining images were matched and aligned manually. During this process, the mismatch of the images to a certain extent was inevitable due to the slight differences between the scan planning by the MRI operator and slices dissection by the pathologist. To match the images perfectly, a study based on *ex-vivo* slices image scan would be required.

## Conclusions

Using a swine model of MI, our study demonstrated that T1 mapping underestimated the size and transmural extent of myocardial infarction relative to histopathology. However, this technique can detect the location of infarction with high specificity. Thus, keeping in mind that it is slightly less sensitive than LGE imaging, native T1 mapping has potential utility as a feasible alternative to LGE imaging in patients with renal insufficiency who cannot receive gadolinium as it can detect location of the infarction with high specificity and give a relatively decent estimate of the amount and extent of the myocardial infarction in a patient group that cannot be imaged with LGE techniques.

## Electronic supplementary material

Supplementary Figure S1
