# Supplementary material for: Detection of Recent Myocardial Infarction Using Native T1 Mapping in a Swine Model: A Validation Study
Source: Sci Rep. 2018 May 9;8:7391. doi: 10.1038/s41598-018-25693-1 (PMC5943450; doi:10.1038/s41598-018-25693-1)

**Detection of Recent Myocardial Infarction Using Native T1 Mapping in a Swine Model: A Validation Study**

Chen Cui^1+^, PhD, Shuli Wang^1+^, MD, Minjie Lu^1,4^*, MD, PhD, Xuejing Duan^2^, MD, Hongyue Wang^2^, MD, Liujun Jia^3^, MD, Yue Tang^3^, MD, Sirajuddin, Arlene^4^, MD, PhD Sanjay K Prasad^5^, MD, PhD, Peter Kellman^6^, PhD, Andrew Arai^4^, MD, PhD, Shihua Zhao^1^*, MD, PhD, FESC, FACC

^1^Department of Magnetic Resonance Imaging, ^2^Department of Pathology, ^3^Department of Animal Experimental Center, Fuwai Hospital, State Key Laboratory of Cardiovascular Disease, National Center for Cardiovascular Diseases, Chinese Academy of Medical Sciences and Peking Union Medical College, Beijing, China

^4^National Heart, Lung and Blood Institute (NHLBI), National Institutes of Health (NIH), Bethesda, Maryland, USA;

^5^NIHR Cardiovascular Biomedical Research Unit, Royal Brompton & Harefield NHS Foundation Trust, London, UK.

^6^Cardiovascular and Pulmonary Branch, National Heart, Lung and Blood Institute, National Institutes of Health, US Department of Health and Human Services, Bethesda, MD, USA

**^*^ corresponding**: lumjcn@hotmail.com; [cjrzhaoshihua2009@163.com](mailto:cjrzhaoshihua2009@163.com)

**^+^ these authors contributed equally to this work**


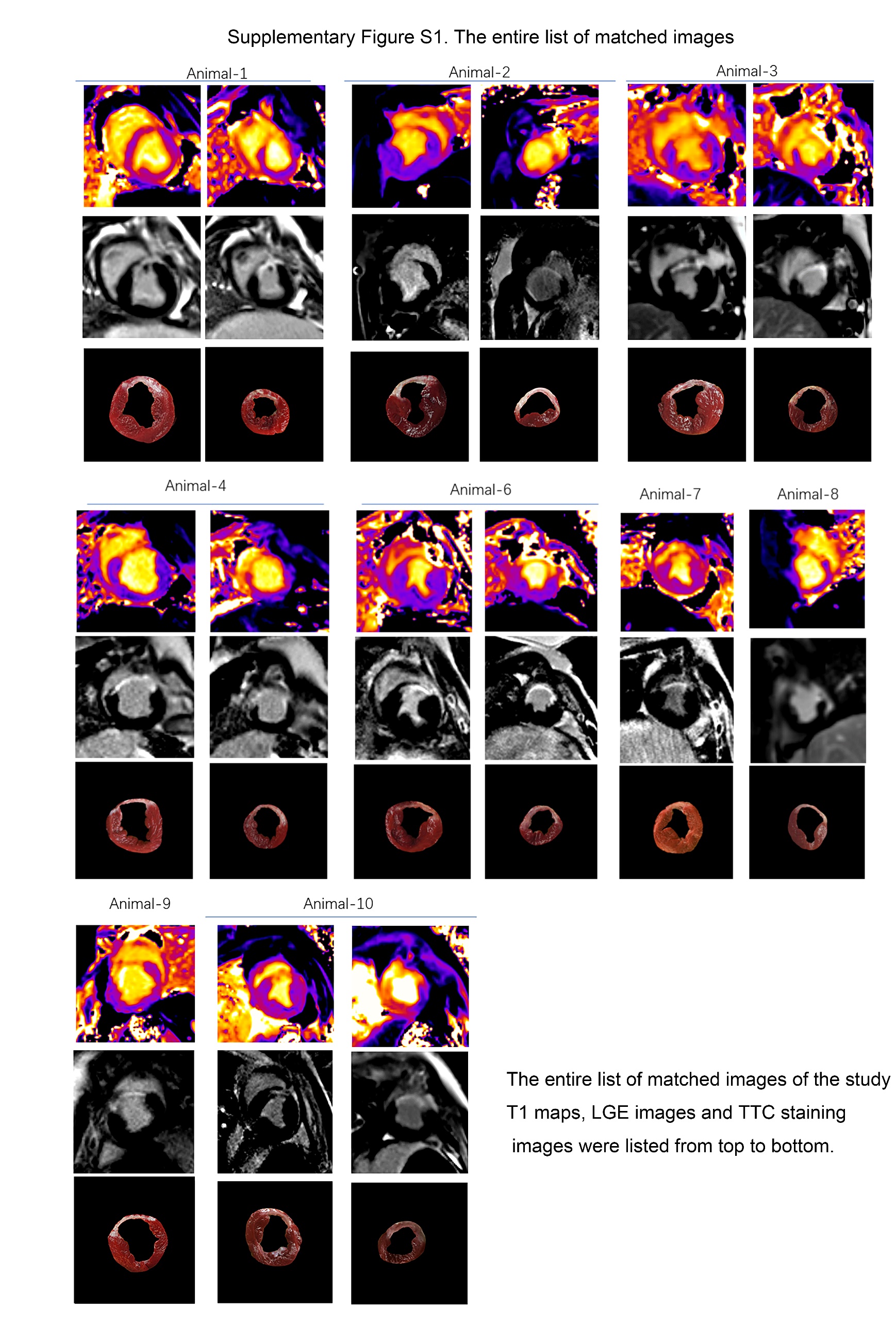

Supplement: Supplementary file 1 — Supplementary Figure S1 [file 41598_2018_25693_MOESM1_ESM.docx]
